# Modulation of apoptosis‐related microRNAs following myocardial infarction in *fat‐1* transgenic mice vs wild‐type mice

**DOI:** 10.1111/jcmm.13846

**Published:** 2018-09-14

**Authors:** Huan Ma, Peipei Chen, Chuanlan Sang, Daozheng Huang, Qingshan Geng, Lei Wang

**Affiliations:** ^1^ Cardic Rehabilitation Department Guangdong General Hospital Guangdong Cardiovascular Institute Guangdong Academy of Medical Sciences Guangzhou China; ^2^ Intensive Care Research Team of Traditional Chinese Medicine 2nd Affiliated Hospital of Guangzhou University of Chinese Medicine Guangdong Provincial Hospital of Chinese Medicine Guangzhou China; ^3^ Laboratory of Experimental Animal Guangzhou University of Chinese Medicine Guangzhou China; ^4^ Intensive Care Unit of Guangdong Geriatric Institute Guangdong General Hospital and Guangdong Academy of Medical Sciences Guangzhou China; ^5^ Guangdong General Hospital Guangdong Academy of Medical Sciences Guangzhou China; ^6^ Department of Cardiovascular Medicine 2nd Affiliated Hospital of Guangzhou University of Chinese Medicine Guangzhou China

**Keywords:** fat‐1, heart, miRNAs, myocardial infarction, omega‐3 polyunsaturated fatty acid

## Abstract

**Background:**

microRNAs (miRNAs) post‐transcriptionally regulate cardiac repair following myocardial infarction (MI). Omega‐3 polyunsaturated fatty acid (ω‐3 PUFAs) may support cardiac healing after MI, but the mechanism is unclear.

**Methods:**

The *fat‐1* transgenic mouse expresses a ω‐3 fatty acid desaturase which converts ω‐6 PUFAs to ω‐3 PUFAs in vivo. MI was induced in *fat‐1* transgenic (n = 30) and wild‐type (WT) mice (n = 30) using permanent ligation. Other transgenic and WT mice underwent sham procedure (n = 30 and n = 30, respectively). One week after occlusion, cardiac function was measured by echocardiography and the infarct size was assessed using histology and miRNA microarray profiling. Expression of selected miRNA was confirmed using quantitative real‐time PCR.

**Results:**

One week following MI, the *fat‐1* transgenic myocardium had better cardiac function, a smaller fibrotic area, and fewer apoptotic cardiomyocytes than WT myocardium. Post‐MI profiling showed 33 miRNAs that were significantly up‐regulated, and 35 were down‐regulated, in *fat‐1* group compared to the WT group (n = 3 and n = 2 mice, respectively). Among selected apoptosis‐associated miRNAs, 9 miRNAs were up‐regulated (miR‐101a‐3p, miR‐128‐3p,miR‐133a‐5p,miR‐149‐5p,miR‐192‐5p,miR‐1a‐3p,miR‐208a‐3p,miR‐29c‐5p,miR‐30c‐2‐3p), and 3 were down‐regulated (miR‐210‐3p,miR‐21a‐3p,miR‐214‐3p) in *fat‐1* transgenic mice compared with WT mice. Kyoto encyclopaedia of genes and genomes (KEGG) pathway analysis indicated likely roles for these miRNAs in MI. Furthermore, Bcl‐2 expression was increased, and caspase‐3 decreased, in infarcted *fat‐1* transgenic mouse hearts compared to WT hearts.

**Conclusions:**

ω‐3 PUFAs may have a protective effect on cardiomyocytes following MI through their modulation of apoptosis‐related miRNAs and target genes.

## 1 INTRODUCTION

Myocardial infarction (MI) is a major cause of morbidity and mortality worldwide. It can lead to loss of cardiomyocytes, left ventricular remodelling, decreased cardiac function, and potentially heart failure.^1^ In recent years, evidence has suggested that omega‐3 fatty acids (ω‐3 PUFAs) have a cardioprotective effect in coronary heart disease and heart failure (HF)^2^, ^3^, ^4^, ^5^; however, the mechanism this protection is not fully understood.

Following MI, cardiomyocyte apoptosis in the border zone around infarct scars and in the remote zone of the noninfarcted myocardium,^6^, ^7^, ^8^ exacerbates remodelling and aggravates cardiac dysfunction.^9^, ^10^ Reversal of cardiomyocyte apoptosis during early stage of MI is crucial for maintaining cardiac function.^11^

Studies have shown that ω‐3 PUFAs can protect cells against apoptosis; including smooth muscle cells,^12^ cardiomyocytes during pressure overload‐induced cardiac hypertrophy,^13^ and neuronal apoptosis following hyperoxic injury.^14^ Therefore, ω‐3 PUFAs may have anti‐apoptotic effects on cardiomyocytes following MI.

microRNAs (miRNAs) are short, noncoding and single‐stranded RNA sequences that typically consist of approximately 18‐22 nucleotides. miRNAs regulate their target genes by binding to the complementary 3' untranslated region (3'UTR) of mRNAs, resulting in translational repression or degradation. Expression of around two‐thirds of all human genes is thought to be regulated by miRNAs. During the last decade, studies have demonstrated that miRNAs modulate various biological functions, including development, differentiation, proliferation, and apoptosis.^15^ They are critically involved in heart function in both physiological and pathophysiological conditions.

The effect of ω‐3 PUFAs on miRNA regulation is not well understood. In this study, we aimed to analyse the miRNA expression profile in the infarcted myocardium of *fat‐1* transgenic and wild‐type (WT) mice to determine whether a lower ω‐6 PUFA/ω‐3 PUFA ratio can alter the expression of miRNAs that regulate cardiomyocyte apoptosis. We propose that higher tissue ω‐3 PUFAs concentrations are cardioprotective effects in MI via the modulation of apoptosis‐related miRNA expression.

## 2 METHODS

### 2.1 Animals

C57BL/6 *fat‐1* transgenic mice were kindly provided by Prof. JX Kang (Massachusetts General Hospital, Harvard University, Boston, MA, USA) and maintained in Guangzhou University of Chinese Medicine. This study was performed in accordance with the approval and guidelines from the Institutional Animal Care and Use Committee of Guangdong Provincial Hospital of Chinese Medicine, Guangzhou University of Chinese Medicine. Mice were housed in a pathogen‐free barrier facility accredited by the Center for Comparative Medicine, and maintained on a 12‐hour dark/night cycle in temperature‐controlled rooms (22.5 ± 0.5°C, 50 ± 5% humidity). Animals had ad libitum access to water and an AIN‐76A/10% corn oil diet (Testdiet, Richmond, IN, USA). This diet is composed of 20% protein, 58% carbohydrate, and 22% fat. It is rich in ω‐6 fatty acids (FAs) and relatively deficient in ω‐3 FAs, mimicking a Western diet. The expression of *fat‐1* in was confirmed by genotyping and tissue FA profiling.

### 2.2 Gas chromatography analysis of FAs

The profile of FAs in the heart was determined by gas chromatography as described previously.^16^, ^17^ In brief, ~10 mg heart tissue was ground into powder under liquid nitrogen, then centrifuged for 5 minutes at 100 *g*. The sediment was retained for FA methylation by 14% boron trifluoride (BF3)–methanol reagent (Sigma‐Aldrich, St Louis, MO, USA) at 100°C for 1 hour. FA methyl esters were analysed with the Agilent HP6890N gas chromatography system equipped with a flame ionization detector (Agilent, Palo Alto, CA, USA).FA peaks were identified by comparing their relative retention times with commercial mixed standards (Nu‐Chek Prep, Elysian, MN, USA), and the area percentages for all resolved peaks were analysed using GC Chemstation software. FA mass was determined by comparing areas of various analysed FAs to that of a fixed concentration of external standard.

### 2.3 Mouse MI model

Male *fat‐1* transgenic and WT mice (200‐250 g) were randomly divided into four groups: WT sham (n = 30), WT MI (n = 30), *fat‐1* sham (n = 30) and *fat‐1* MI (n = 30).

All mice were heparinized (300 U), anesthetized with ip, sodium pentobarbital (40 mg/kg), and ventilated using a small animal ventilator at a frequency of 120/min and tidal volume of 0.8 mL. Body temperature was maintained at 37°C using a heating pad while each chest was surgically opened along the midline. A standard limb lead ECG continuously recorded while a left thoracotomy was performed at the 4th rib and a segment of saline‐soaked 7‐0 sutures was looped around the left anterior descending (LAD) coronary artery, near its origin from the left coronary artery. The ends of the suture were passed through a 1‐inch segment of double‐barrelled polyethylene tubing. The ends of the tubing were rounded so that a smooth surface abutted the coronary artery.

In mice from the MI groups, the tubing was gently placed next to the heart and secured with 3 suture knots, with the last knot up against the ends of the tubing. The tubing and suture assembly were then externalized and the thorax closed. Occlusion was accomplished by withdrawing the distal knot 2 mm away from the tubing end. This permits reproducible occlusion of the LAD without tearing the artery. Successful occlusion was confirmed by the increase in the amplitude of the R wave of lead I during the first few seconds of each occlusion, and elevation of ST segment of lead II. Mice from the sham group underwent the same procedure but the LAD was not occluded.

### 2.4 Echocardiography

Post‐MI left ventricular function was measured after 1 week using the Visual SonicsVevo^®^ 2100 small animal ultrasound system (VisualSonicsInc, Toronto, ON, Canada). Left ventricular systolic diameter (LVDSd) and left ventricular diastolic diameter (LVDDd) was measured, while left ventricular ejection fraction (LVEF) and fractional shortening (FS) were calculated.

### 2.5 Histology

Following the post‐MI echocardiography, mice were humanely killed. The hearts were harvested and 6 from each group were fixed in 4% paraformaldehyde. Fixed hearts were sectioned into 5‐μm‐thick sections with longitudinal cutting, then stained with haematoxylin and eosin or Masson's Trichrome.

Using computer‐based planimetry, infarct size was calculated as the sum of the infarcted epicardial and endocardial circumferences divided by the sum of the total left ventricular epicardial and endocardial circumferences. Quantitative assessment of each parameter was performed using image analysis software (OlympusBX41+ DP25, Olympus, Tokyo, Japan).

For apoptosis analysis, terminal deoxynucleotidyl transferase‐mediated dUTP nick end‐labelling (TUNEL) was performed using an in situ Cell Death Detection Kit (Roche AppliedScience, Penzberg, Germany), according to the manufacturer's instructions.

Cleaved caspase3 (c‐caspase 3), bax, Bcl‐2, and proliferating cell nuclear antigen (PCNA) (all from Sigma‐Aldrich Co. LLC) immunochemical staining was also conducted according to the manufacturer's instructions.

### 2.6 RNA isolation

The infarcted parts of myocardium, the border zone of infarct scars, and the remote zone of noninfarcted myocardium from infarcted *fat‐1* transgenic mice (n = 3) and infarcted WT mice (n = 3) were used for RNA (50%) and protein extraction (50%).

Total RNA was isolated using kits according to manufacturer's instructions. For detecting the microRNA expression, miRcute miRNA isolation kit, miRcute miRNA First‐Strand cDNA synthesis kit and miRcute miRNA qPCR detection kit (SYBR Green) were used (all from Vazyme Biotech Co. Ltd, Nanjing, China). For detecting mRNA, TRIzol RNA (Invitrogen, Carlsbad, CA, USA), ThermoScript™ RT‐PCR system and a Fast Start universal SYBR green master (ROX) were used. RNA quality and integrity were determined using a 2100 bioanalyzer (Agilent Technologies Inc) and RNA LabChip^®^ kits (Agilent Technologies Inc).

### 2.7 miRNA arrays

Small RNAs were isolated from the total RNA and labelled with Cy3. The Oebiotech Company performed the miRNA microarray assay. The fragmentation mixtures were hybridized to an Agilent‐046065 mouse miRNA microarray V19.0 8 × 60K (Agilent). Feature Extraction software 10.7.1.1 (Agilent) analysed the scanned images using default parameters to obtain background subtracted and spatially de‐trended the processed signal intensities as the raw data. Raw data were normalized using a quantile algorithm with GeneSpring 12.5 (Agilent) Probes for which where all samples in any 1 condition out of 2 conditions had flags in “Detected” were maintained. The raw data of the microarray have been uploaded to GEO with the series record GSE57127. Values < 0.01 were set to 0.01 and each measurement was divided by the 75th percentile of all measurements from the same samples. miRNAs whose expression differed by at least 2‐fold between *fat‐1* transgenic MI and *WT* MI groups were selected for further investigation.

### 2.8 miRNA target predictions

miRNA targets were predicted using TargetScan, PITA, and microRNA.org databases. Fifteen universally predicted apoptosis‐associated miRNAs formed the panel for this study:miR‐1a‐3p, miR‐21a‐3p, miR‐24‐3p, miR‐29c‐5p, miR‐30c‐2‐3p,miR‐101a‐3p,miR‐126a‐3p,miR‐128‐3p,miR‐133a‐5p,miR‐149‐5p,miR‐192‐5p, miR‐208a‐3p, miR‐210‐3p,miR‐214‐3p and miR‐299‐5p.Kyoto encyclopaedia of genes and genomes (KEGG) pathway analysis was used to assess the roles of these miRNAs.

### 2.9 Quantitative real‑time PCR analysis

Following MI, total cellular RNA was extracted using Trizol reagent (Invitrogen) from 6 *fat‐1* transgenic and 6 WT hearts. For miRNA analysis, mature miRNA was reverse‐transcribed from total RNA using a TaqMan microRNA reverse transcription kit. Real‐time PCR was performed according to the manufacturer's instructions. miRNA expression was determined using the 2^(−▵▵Ct)^ method and normalized against small nuclear RNA (snRNA) U6.

For quantification of c‐caspase‐3, bax, bcl‐2 and PCNA, cDNA was generated using a Prime Script RT reagent Kit (TaKaRa; Dalian, China), and real‐time PCR was performed on an ABIPrism 7000 sequence detection system with SYBR premix ex Taq (TaKaRa). All experiments were performed 3 times, in triplicate.

### 2.10 Western blot analysis

Mouse heart tissue samples were homogenized in modified RIPA buffer. Protein samples were mixed with sample buffer, boiled for 10 minutes, separated by SDS‐PAGE under denaturing conditions, and electroblotted to nitrocellulose membranes. Blots were incubated overnight in Tris‐buffered saline containing 5% skim milk to block nonspecific binding of the antibody. Proteins of interest were revealed with specific antibodies (rabbit monoclonal antibody to Caspase 8 Associated Protein 2 [CASP8AP2], Abcam Biotechnology, Shanghai, China) as indicated (1:1000 dilution) for 1 hour at room temperature followed by incubation with a 1:5000 dilution of horseradish peroxidase‐conjugated polyclonal anti‐rabbit antibody for 1 hour at room temperature. Signals were visualized by chemiluminescent detection. Equal protein loading of samples was further verified by the staining monoclonal antibody, GAPDH. All Western blots were quantified using densitometry.

### 2.11 Statistical analyses

Values were expressed as the mean ± SEM and statistical analyses were conducted using SPSS software. A *t*‐test was used to compare miRNA and mRNA expression between *fat‐1* transgenic MI and WT MI groups. One‐way ANOVA was used to compare cardiac function (LVEF and FS), Masson stain area, and the density of apoptotic cardiomyocytes between groups. The results were considered significant if *P *<* *0.05. Statistical analysis for miRNA is described in the methodological section for each array.

## 3 RESULTS

### 3.1 *Fat‐1* transgenic mice phenotype identification

The ratio of omega‐6 fatty acids (ω‐6 PUFAs)/ω‐3 PUFAs was around 1.3 in *fat‐1* mice and approximately 9.4 in the WT group (Table 1).

**Table 1:** The cardiac fatty acid profile in *fat‐1* transgenic mice (n = 3) and WT mice (n = 3)

	WT	Fat‐1
Omega‐6	5.56 ± 0.51	3.35 ± 0.80^a^
Omega‐3	0.68 ± 0.29	2.66 ± 0.70^a^
Omega‐6/Omega‐3	9.49 ± 0.38	1.32 ± 0.36^a^

a *P* < 0.05.

### 3.2 Effect of a low ω‐6 PUFA/ω‐3 PUFA ratio on post‐MI cardiac function

**Figure 1:**
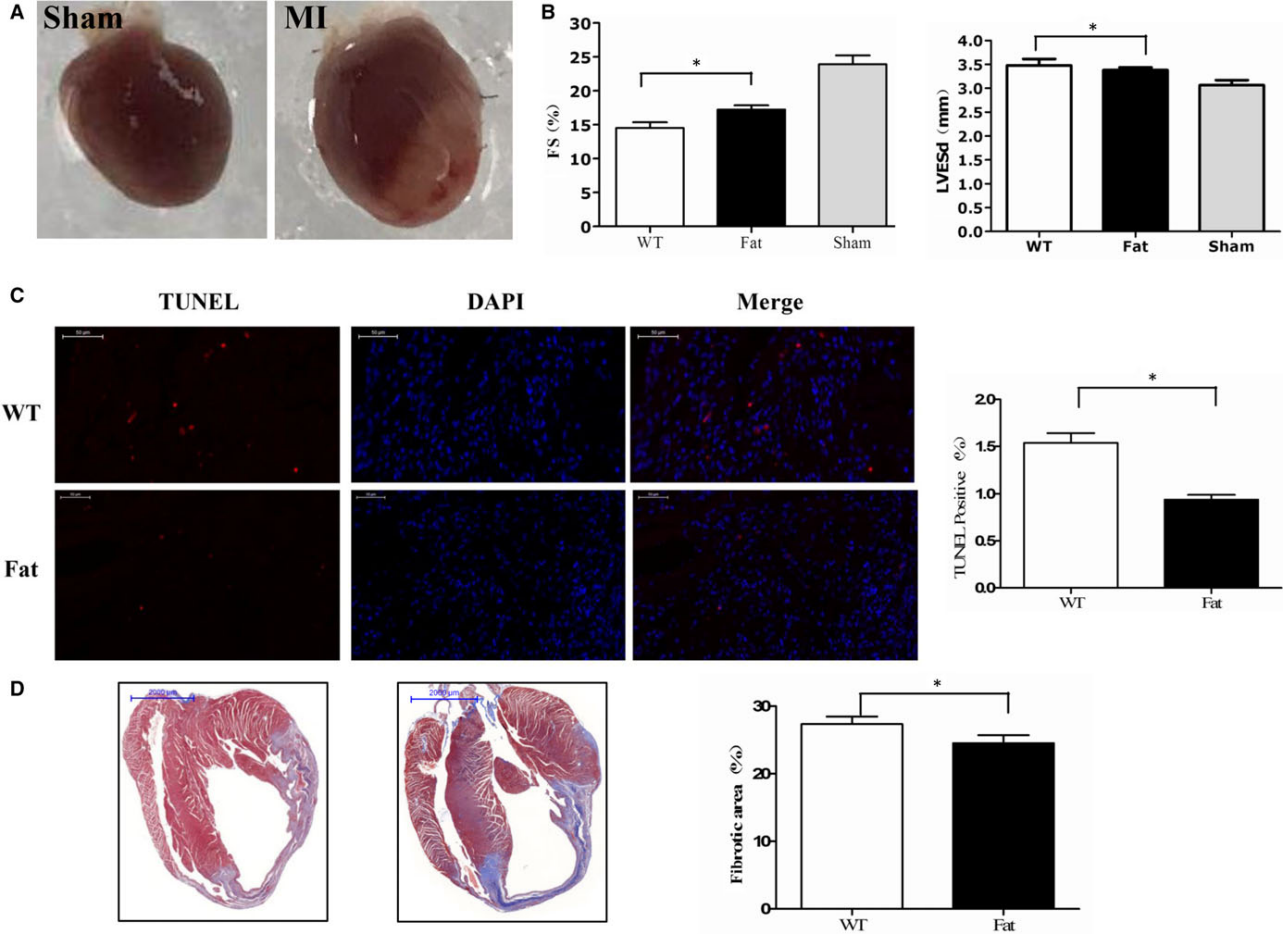
Cardiacfunction, myocardial fibrosis and cardiomyocyte apoptosis, 1 wk after myocardial infarction (MI) in *fat‐1* transgenic and wild‐type mice. A, Hearts isolated from sham and MI‐treated mice. B, Cardiac function assessed by echocardiography; *P < 0.05. C, Representative histological sections of the infarcted myocardium stained with TUNEL assay. Quantitative analysis was performed for TUNEL positive cells. Scale bar = 50 μm; *P < 0.05. D, Fibrosis detected with Masson's trichrome staining from 6 mice per group, Scale bar = 2 mm; *P < 0.05

To determine whether ω‐3 PUFAs have a cardioprotective effect on the ischaemic myocardium, cardiac function was analysed by echocardiography, 1 week after MI. *Fat‐1* transgenic mice showed a significantly increased FS, LVEF, and a decreased left ventricular end‐diastolic dimension (LVEDd) compared with WT mice(Figure 1B).

Masson's Trichrome staining showed a significantly smaller fibrotic area in *fat‐1* transgenic hearts compared with WT hearts, post‐MI (Figure 1D). Figure 1C also shows that there were fewer apoptotic cardiomyocytes in the myocardium.

### 3.3 A low ω‐6PUFA/ω‐3 PUFA ratio has anti‐apoptotic effects in the infarcted myocardium

**Figure 2:**
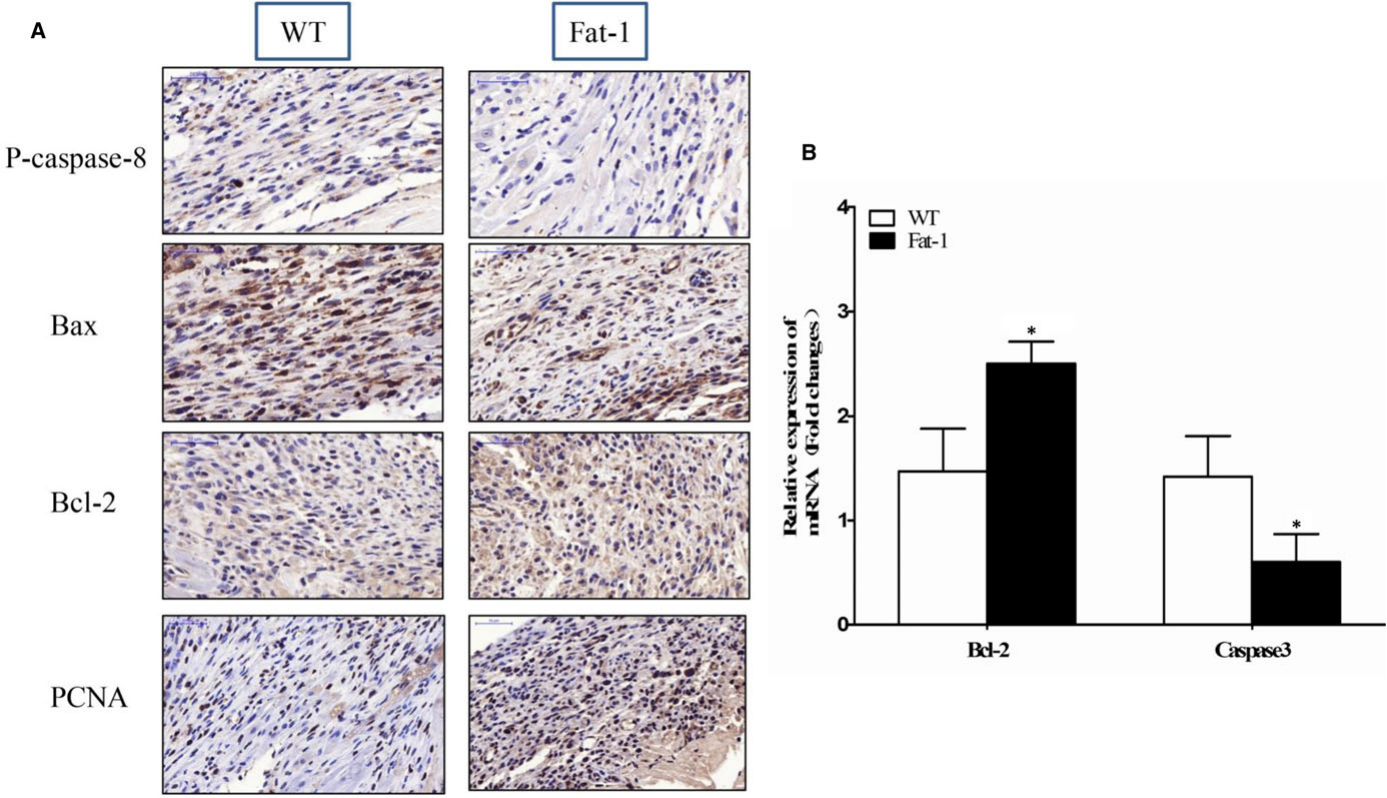
mRNA and protein expression of apoptotic markers in the infarcted area, 1 wk after myocardial infarction in *fat‐1* transgenic and wild‐type mice. Assessed by immunocytochemical staining and quantitative real‐time PCR; *P < 0.05

C‐caspase 3 is a key factor in the apoptosis pathway and its expression level is positively correlated with apoptosis. C‐caspase 3 protein, detected using immunocytochemistry, was lower in cardiac tissue from *fat‐1* transgenic mice compared with WT mice (Figure 2A). The mRNA expression of caspase‐3 (determined by quantitative PCR) showed a comparable result and was statistically significant (Figure 2B).

The amount of Bax, Bcl‐2, and PCNA protein were also determined by immunohistochemical staining. Following MI*, fat‐1* transgenic mice had more of Bcl‐2 protein in their cardiac tissue than WT mice (Figure 2B).

### 3.4 Infarcted myocardium miRNA profile

Three infarcted myocardium samples from *fat‐1* transgenic mice and 2 from WT mice were used the miRNA array. The sample from 1 WT mouse was excluded because of poor RNA quality.

**Figure 3:**
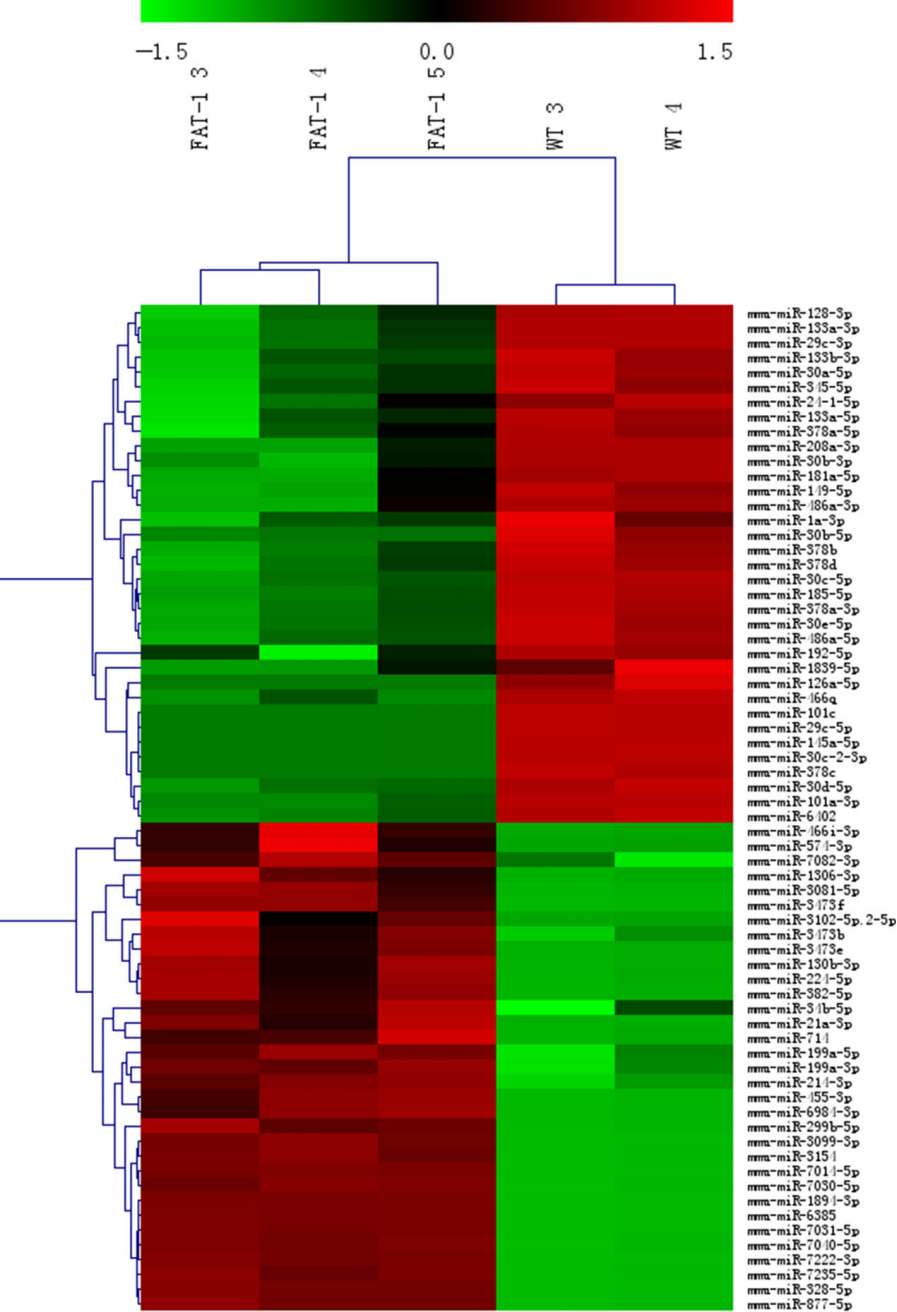
Heat map of differentially expressed microRNAs (miRNAs) between the infarcted myocardium of *fat‐1* transgenic mice (n = 3) and wild‐type (WT) mice (n = 2). Fold change > 2. Each row represents one miRNA and each column represents a myocardium sample. The legend on the right indicates the miRNA represented in the corresponding row. The relative miRNA expression is depicted according to the colour scale; red indicates up‐regulation; green indicates down‐regulation. The mark with FAT‐1 indicates *fat‐1* transgenic mice, the mark with WT indicate WT mice

Sixty‐eight miRNAs were differentially expressed between the transgenic and WT groups, including 33 that were up‐regulated and 35 that were down‐regulated (more than a 2‐fold change; *P *<* *0.05). In unsupervised hierarchical clustering analysis, normalized microarray expression for the 68 miRNAs showing differential expression in the five infarcted myocardium samples were used to generate a heat map (Figure 3).

### 3.5 Analysis of infarcted myocardium miRNAs by quantitative real‐time PCR

Following the selection strategy, fifteen miRNAs were chosen to be verified by quantitative real‐time PCR (Table 2).

**Table 2:** microRNAs with apoptosis characteristics identified from the microarray analysis of infarcted myocardium from *fat‐1* transgenic (n = 3) and wild‐type mice (n = 2). (>2‐fold change)

Gene name	Fold change	*P*‐value	Regulation
mmu‐miR‐1a‐3p	3.71	0.029	Down
mmu‐miR‐21a‐3p	15.46	0.014	Up
mmu‐miR‐24‐1‐5p	7.12	0.048	Down
mmu‐miR‐29c‐5p	19.64	0.002	Down
mmu‐miR‐30c‐2‐3p	53.83	4.94E‐08	Down
mmu‐miR‐101a‐3p	2.33	4.62E‐04	Down
mmu‐miR‐126a‐3p	27.26	0.002	Down
mmu‐miR‐128‐3p	5.68	0.018	Down
mmu‐miR‐133a‐5p	6.334	0.028	Down
mmu‐miR‐149‐5p	6.86	0.037	Down
mmu‐miR‐192‐5p	14.37	0.048	Down
mmu‐miR‐208a‐3p	72.5391	0.015	Down
mmu‐miR‐210‐3p	4.841915	0.014	Up
mmu‐miR‐214‐3p	2.27	0.002	Up
and miR‐299‐5p	19.64	0.002	Up

**Figure 4:**
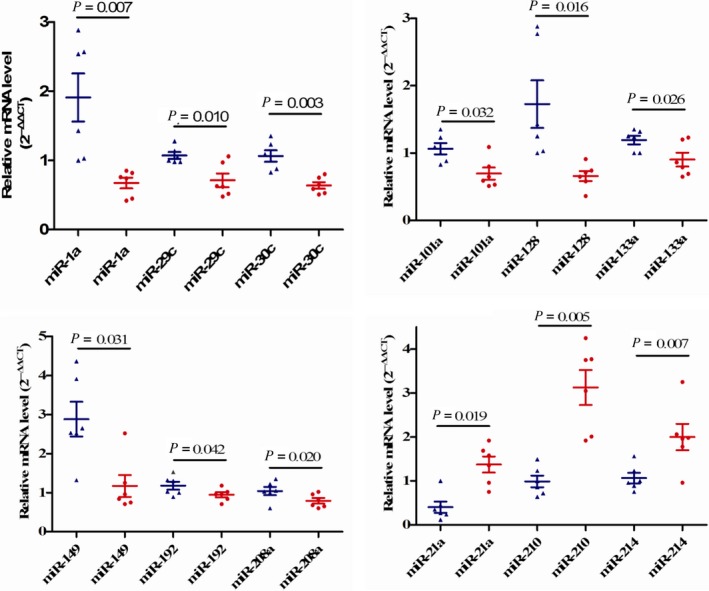
Validation of microRNA (miRNA) expression by quantitative real‐time PCR. Analysis of the infarcted myocardium from *fat‐1* transgenic and wild‐type (WT) mice (n = 6 each). miRNA expression data are presented as fold change relative to the WT group. Data are represented by scatter plots where line represents the median value. The *P*‐values were calculated by *t*‐test. Blue indicates WT mice group. Red indicates *fat‐1* group

Most results were consistent with microarray data. Twelve miRNAs showed statistically significant differences in expression between infarcted *fat‐1* transgenic hearts and infarcted WT hearts; miR‐1a‐3p, miR‐29c‐5p,miR‐30c‐2‐3p,miR‐101a‐3p, miR‐128‐3p,miR‐133a‐5p,miR‐149‐5p,miR‐192‐5p, and miR‐208a‐3p had lower expression in *fat‐1* transgenic hearts; MiR‐210‐3p, miR‐21a‐3p, and miR‐214‐3p had higher expression in *fat‐1* transgenic hearts (Figure 4).

### 3.6 The time‐course trend of miR‐210 expression and its target gene

**Figure 5:**
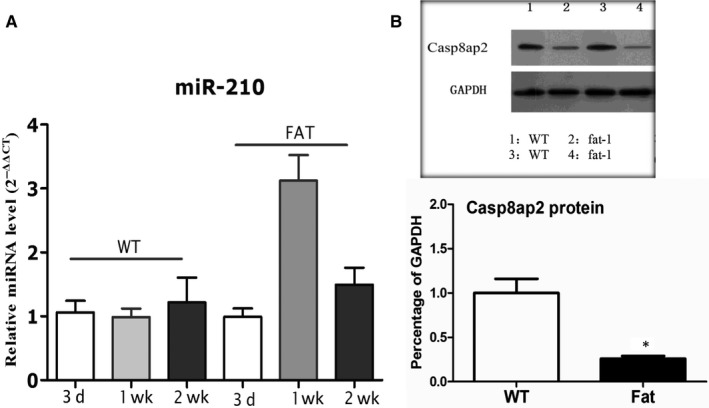
Time‐course trend of miR‐210 expression and the protein level of CASPASE8AP2 in infarcted heart. A, The expression level of miR‐210 in WT or fat‐1 mice (n = ? per group) at 3, 7 and 14 d after myocardial infarction were detected by qPCR. B, the protein level of Caspase8ap2 in heart tissues of *fat‐1* and WT groups was analysed by Western blotting at week‐1 post‐MI

To test the time course of miR‐210 expression, expression level of miR‐210 in heart tissue was measured at 3, 7 and 14 days after myocardial infarction. It showed that miR‐210 was up‐regulated in the *fat‐1* mice as compared to WT mice at week‐1 post‐MI (Figure 5A). The protein level of CASPASE8AP2, a target gene of miRNA‐210, was also measured. Western blotting results showed that the protein level of Caspase8ap2 in *fat‐1* group is lower than in WT group. CASPASE8AP2 is known to promote cardiomyocyte apoptosis, thus the reduction of CASPASE8AP2 level may prevent cardiomyocyte apoptosis.

### 3.7 Apoptosis pathway analysis of 12 different expressed miRNAs

ω‐3 PUFAs are reported to have protective effects on cells in many common disease, such as cardiovascular diseases, metabolic diseases, and cancers; many potential mechanisms have been proposed.^18^, ^19^, ^20^ We analysed the target genes of the 12 differentially expressed miRNAs to further explore the effects of ω‐3 PUFAs.

**Figure 6:**
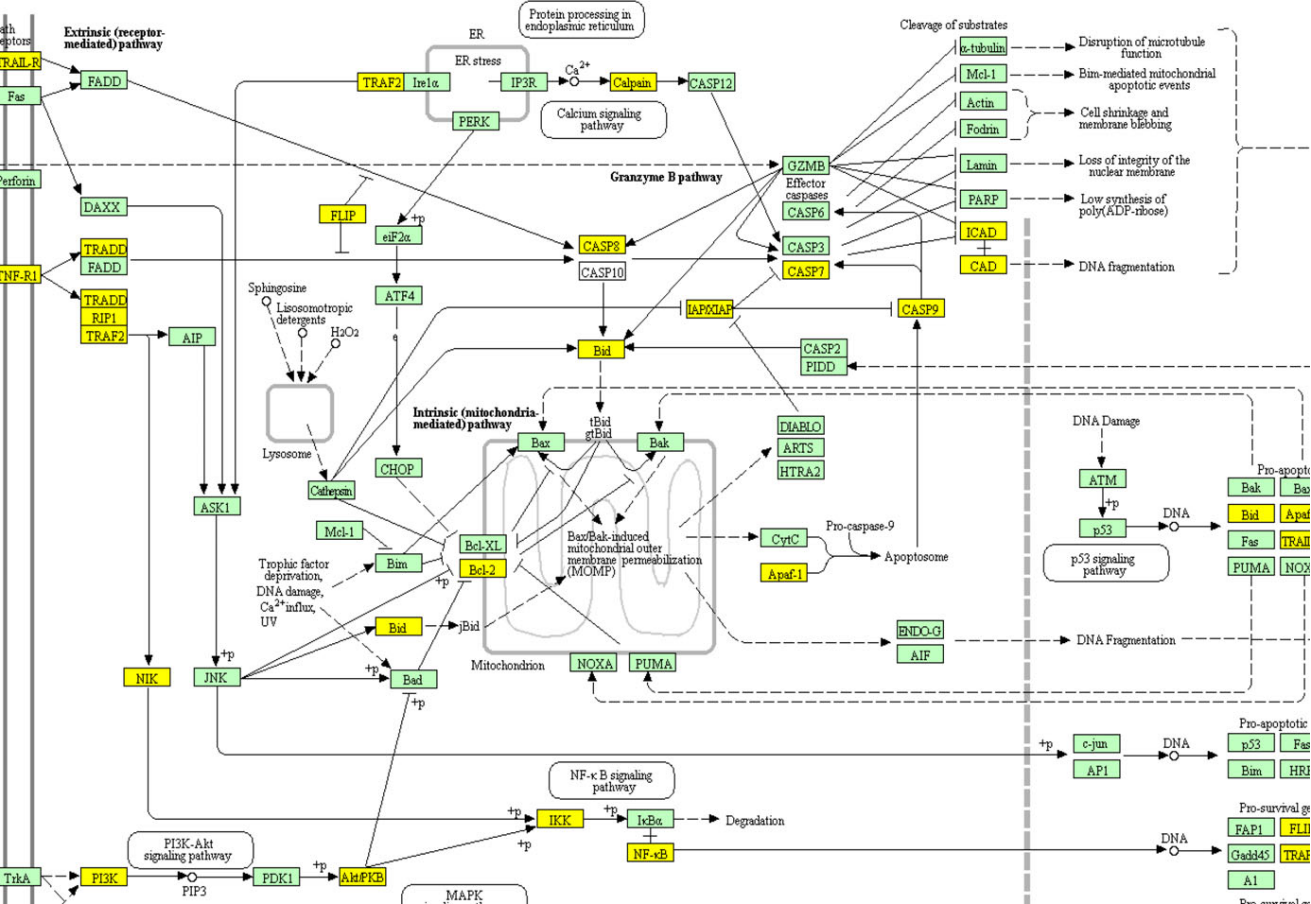
Apoptosis pathway analysis for the targets of 12 different expressed microRNAs (miRNAs) in infarcted myocardium of *fat‐1* transgenic vs wild‐type mice. Target genes were highlighted in apoptosis pathways from the Kyoto encyclopaedia of genes and genomes (KEGG) map. The yellow highlighted genes were known or predicted targets of one miRNA, and the brown genes were targeted by more than one miRNA

Most of the gene targets were related to apoptosis, as shown in Figure 6. The genes highlighted in yellow and brown were predicted targets in the apoptosis pathway, identified in the KEGG map. For example, miR‐210 is thought to inhibit the expression of caspase‐8 associated protein 2 (CASPASE8AP2), which, consequently inhibits the expression of caspase‐8 and myocardium apoptosis.

## 4 DISCUSSION

Our study utilized *fat‐1* transgenic mice to demonstrate the protective effects of a low cardiac tissue ω‐6 PUFA/ω‐3 PUFA ratio in mice undergoing MI. Following MI, *fat‐1* transgenic mice had better cardiac function, less cardiac fibrosis, and fewer apoptotic cardiomyocytes. Protein, mRNA and miRNA analyses showed that infarcted cardiac tissue from *fat‐1* transgenic mice was undergoing less apoptosis at 1‐week post‐MI.

In this study, we were investigating the potential health benefits of ω‐3 PUFAs and took advantage of the *fat‐1* transgenic mouse, which carries the caenorhabditis elegans fat‐1 gene encoding an ω‐3 FA desaturase. This enzyme converts ω‐6 PUFAs to ω‐3 PUFAs by adding a double bond at the ω‐3 position^21^; therefore, *fat‐1* transgenic mice have a much lower ω‐6/ω‐3 PUFA ratio in their tissues and organs (~1), than WT mice (~9). As this is a transgenic mouse line, it is possible to create individuals with very different FA profiles within the same litter, while maintaining an identical diet. Hence, the *fat‐1* mouse is a useful tool for elucidating the effects of a low ω‐6 PUFA/ω‐3 PUFA ratio in vivo.

Analyses of the differentially expressed miRNAs in infarcted cardiac tissue from *fat‐1* transgenic and WT mice was conducted to try and decipher the complex influence of ω‐3 PUFAs in MI and to identify putative cardioprotective genes. A subset was confirmed by real‐time PCR analysis. We found significant differences in the expression of apoptosis‐related miRNAs and their predicted target mRNAs in the infarcted myocardium of *fat‐1* transgenic mice compared with WT mice.

We identified 9 apoptotic‐related miRNAs that had a higher expression in infarcted myocardium from *fat‐1* transgenic mice than in WT mice, while 3 had a lower expression. Each is believed to be cardiac‐specific or cardiac‐enriched miRNAs involved in differentiation of heart myocytes and maintaining the functionality/survival of cardiac muscle cells.^22^ The activity and function of these miRNAs are strictly regulated to ensure proper cardiac contractility and conduction. In pathological conditions such as MI, deregulation of these miRNAs may lead to myocardial apoptosis, necrosis, fibrosis and other destructive processes, eventually lead to cardiac arrhythmia, hypoxia, ischaemia, left ventricular dilatation. Modulation of specific miRNAs could serve as potential therapeutic approaches for MI.^23^

In this study, we saw that *fat‐1* transgenic mice (which have a lower ratio of ω‐6 PUFA/ω‐3 PUFAs in the myocardium) are partially protected from MI‐induced cardiomyocyte apoptosis. This was correlated with a specific miRNA expression profile. miRNA expression profiles play an essential role in apoptotic‐related signalling pathways such as phosphoinositide 3‐kinase (PI3K), Bcl‐2 and caspase pathways. The Bcl‐2 family of proteins includes anti‐apoptotic members like Bcl‐2 and Bcl‐x_L_ along with pro‐apoptotic member Bax, and together regulate mitochondrial integrity during an apoptotic insult. MI‐related cardiomyocyte apoptosis has been shown to involve increased expression of Bax and decreased levels of Bcl‐2.^24^ Our results provide early evidence that lowering the ω‐6/ω‐3 PUFAs ratio can influence apoptosis at the mitochondrial level of by restoring the anti‐apoptotic balance of Bcl‐2. Similarly, another study has found that ω‐3 PUFAs are anti‐apoptotic in developing cerebellum through Bcl‐2 and MAPK pathways.^25^

One of the key events in apoptosis is activation of caspase‐3, and its expression is positively correlated with apoptosis.^26^ Caspase‐3 mRNA and protein expression were decreased in the infarcted myocardium of *fat‐1* mice compared with the infarcted myocardium of the WT group. In this study, we also measured the expression level of miR‐210 at 3, 7 and 14 days after myocardial infarction. It showed that miR‐210 was mostly up‐regulated in the *fat‐1* mice as compared to WT mice at week‐1 post‐MI. The protein level of one target of miRNA‐210—‐ CASPASE8AP2 was lower in *fat‐1* group than in WT group. CASPASE8AP2 is known to promote cardiomyocyte apoptosis, thus the reduction of CASPASE8AP2 level may prevent cardiomyocyte apoptosis. These findings suggest that lowering the ω‐6/ω‐3 PUFA ratio can have an inhibitory effect on caspase‐3 and this may explain some of its cardioprotective effects in MI.

Overall, our study suggests that a lower ω‐6 PUFA/ω‐3 PUFA ratio in the heart can alter the miRNA, mRNA, and ultimately the protein expression profile in the murine infarcted myocardium. Our results suggest that this profile is anti‐apoptotic and results in fewer cardiomyocyte deaths, less fibrosis and better cardiac function. Further research into the effects of the identified miRNA may provide future therapeutic targets to reduce the cardiac damage, morbidity, and mortality caused by MI.

## CONFLICT OF INTEREST

None declared.
